# Quantitative measurement of post-concussion syndrome Using Electrovestibulography

**DOI:** 10.1038/s41598-017-15487-2

**Published:** 2017-11-27

**Authors:** Abdelbaset Suleiman, Brian Lithgow, Zeinab Dastgheib, Behzad Mansouri, Zahra Moussavi

**Affiliations:** 1 Biomedical Engineering Program, University of Manitoba, Winnipeg, MB Canada; 2 Department of Internal Medicine (Neurology), University of Manitoba, Winnipeg, MB Canada; 3 Monash Alfred Psychiatry Research Center, Monash University, Melbourne, Australia

**Keywords:** Brain injuries, Biomedical engineering

## Abstract

In this study, a noninvasive quantitative measure was used to identify short and long term post-concussion syndrome (PCS) both from each other and from healthy control populations. We used Electrovestibulography (EVestG) for detecting neurophysiological PCS consequent to a mild traumatic brain injury (mTBI) in both short-term (N = 8) and long-term (N = 30) (beyond the normal recovery period) symptomatic individuals. Peripheral, spontaneously evoked vestibuloacoustic signals incorporating - and modulated by - brainstem responses were recorded using EVestG, while individuals were stationary (no movement stimulus). Tested were 38 individuals with PCS in comparison to those of 33 age-and-gender-matched healthy controls. The extracted features were based on the shape of the averaged extracted field potentials (FPs) and their detected firing pattern. Linear discriminant analysis classification, incorporating a leave-one-out routine, resulted in (A) an unbiased 84% classification accuracy for separating healthy controls from a mix of long and short-term symptomatology PCS sufferers and (B) a 79% classification accuracy for separating between long and short-term symptomatology PCS sufferers. Comparatively, short-term symptomatology PCS was generally detected as more distal from controls. Based on the results, the EVestG recording shows promise as an assistive objective tool for detecting and monitoring individuals with PCS after normal recovery periods.

## Introduction

Mild traumatic brain injury (mTBI), also known as concussion, is defined as a transient alteration of brain function caused by a biomechanical force to the brain after a head injury, which is usually followed by some permanent or transient neurological symptoms and signs. mTBI is more frequent in teenagers, young adults, males and people who are engaged in high impact physical activities (e.g. soldiers, contact sports players)^1,2^. Following an mTBI, patients usually experience one or more of the following clinical symptoms: headaches, dizziness, depression, memory loss, confusion, blurred vision and balance problems that may occur with or without loss of consciousness^3,4^. The persistence of these symptoms for more than one week is usually referred to as post-concussion syndrome (PCS)^5^. These symptoms except headache and dizziness are often reported for a few days and up to weeks following the injury. The headache and dizziness symptoms typically occur immediately as well as later in the recovery period. The observable neural dysfunction resulting from a head injury in most cases is temporary^3^; however, the symptoms can last for days, weeks (short-term PCS (SPCS)), and sometimes much longer with persisting symptoms^6^ which is referred to as long-term PCS (LPCS)^7^. Permanent, undiagnosed and untreated symptoms may disable the affected person and; in some cases, it may be fatal^8^.

Persistent PCS has been reported in 20–30% of concussed individuals and comprise incomplete recovery^9^ which include somatic (e.g. headaches, dizziness), cognitive (e.g. poor concentration), mood (e.g. depression), visual (e.g. convergence insufficiency, poor accommodation) and behavioral (e.g. irritability) problems^9,10^. These symptoms can be dominant in the first few hours or days right after the impact but may also persist for weeks, months or even years^10^. Neuropsychological assessments following a concussion have shown that the cognitive function mostly recover within 1–3 months’ post-injury^11^. Additionally, some improvements can take place during the first two years but some patients may remain impaired longer than 2 years^11^.

The major problems confounding treatment for concussed patients derive from the fact that there is no universally and clinically accepted tool^12^ with (A) the ability to early detect and diagnose the presence of PCS soon after an impact, or (B) the ability to monitor full recovery.

The most commonly and traditionally applied diagnostic tools for concussion and PCS, such as the Sports Concussion Assessment Tool (SCAT3)^13^, Balance Error Scoring System (BESS)^14^, and computerized cognitive tests such as CogSport^15^ and ImPACT^16^ are subjective, and rely on self-reported symptoms. Other objective tools, like most of the brain imaging techniques, i.e. computed tomography (CT) or susceptibility weighted imaging (SWI) - magnetic resonance imaging (MRI)^17^, cannot reliably diagnose mTBI. In addition, the more reliable ones such as diffuse tensor imagining (DTI) have several limitations (e.g. cost, availability, resolution, etc. ref.^13^). These imaging techniques are more reliable for detecting lesions as the case in SWI-MRI^17^ or skull fracture by CT^13^. However, in the majority of patients with PCS the clinical neuroimaging findings are found to be normal^18,19^. Such disagreement between the imaging finding and the existence of cognitive impairments in PCS group might be due to a damage in the neural network rather than a focal lesion site likely to be detected by imaging techniques.

Neuropsychological assessments after mTBI, on the other hand, have a limited ability to accurately detect the presence of PCS mainly due to the fact that the scores can also be affected by several other confounding factors such as intelligence, age, education, depression and malingering^20^. Thus, having an objective and reliable assessment technique for PCS, such as the one investigated in this study, would be of great interest. There have been some quantitative approaches, such as quantitative EEG (qEEG)^21–23^ and robotic-assisted test battery^24^, investigating PCS and its recovery with some very positive outcomes including, when studying different post-concussion times using qEEG, a reported 77.8–92.3% accuracy in detecting short-term and long-term TBI^23^. However, there are also some studies that question qEEG’s clinical usefulness^12,25^ but recent publications do continue to support its utility^26,27^. The search for a universally clinically acceptable, quantitative PCS assessment tool continues.

Electrovestibulography (EVestG)^28^ has shown promise as a diagnostic assist tool for neurological and neuropsychiatric conditions such as Meniere’s^29^, Depression^30^ and Parkinson’s Disease^31^. EVestG signals are recorded in the external ear. The recorded signal is a combination of acoustic and vestibular generated field potentials (FPs)^28^. The EVestG technology can detect the vestibular response, which is hypothesized to be driven predominantly from the utricle^28^. The utricle has tonically active (spontaneously firing) hair cells that can be modulated by linear acceleration^32–34^. There is support for a vestibular change following mTBI. One of the major symptoms after mTBI is dizziness with about 23–81% of mTBI cases experiencing balance problems and reporting dizziness days after injury^35^. The prevalence of persistent dizziness after mTBI varies widely from 1.2% at 6 months to 32.5% at 5 years^36–39^. EVestG signal analysis can detect the changes in the balance/vestibular system^28^. Using DTI, the vestibulopathy in mTBI patients was found to have a central axonal injury component^40^. In addition, vascular compression of the 8^th^ cranial nerve has been shown to lead to dysfunction of the auditory and vestibular systems^41^. Herein, this study presents staged research to evaluate EVestG as an assistive tool to measure and monitor both short and long-term symptoms of PCS.

We hypothesize that one of the many reasons that mTBI patients experience balance problems is due to stretching, compressing or twisting of the vestibular nerve leading to a change in the vestibular response, which may also be persistent. Likewise, if the damaged regions of the brain that are bruised, swollen or have bled are connected to the vestibular system, their electrical signals related to balance might have changed. Further, during the metabolic cascade following mTBI, the sensory systems including the peripheral vestibular system will be impacted. Thus, the EVestG signals will likely change following a concussive impact, and that may prove to be indicative of a concussion and its continuing symptomatology. This paper reports on the use of EVestG addressing the above hypotheses.

The objectives of this study were to 1) investigate the EVestG detected spontaneous FP changes between concussed individuals with PCS and healthy controls, and 2) investigate whether the features representing those changes have diagnostic classification power between the two groups of PCS and controls.

To accomplish the first objective, we recorded EVestG signals from a group of concussed individuals with PCS as well as a control group of age and gender matched healthy individuals. We compared the extracted field potentials (FPs) from the EVestG signals of the two groups. To achieve the second objective, we extracted two characteristic features that had shown sensitivity to PCS in our previous study^42^ followed by an unbiased classification routine to investigate whether the features have a diagnostic classification power.

## Results

All patients who presented to neuro-ophthalmology clinic and were diagnosed with PCS, met the inclusion criteria for the study and did not have the exclusion criteria, were referred to the research assistant to be included in this study. All referred patients had diagnosis of PCS, which was made based on the history and the positive examination findings such as convergence insufficiency and abnormal balance, determined by the neuro-ophthalmologist collaborator of the study (4^th^ author)^43^.

The inclusion criteria for PCS group were: 1) being over 15 years of age, 2) having at least one head trauma with or without loss of consciousness in the last 10 years, 3) having a Glasgow Coma Scale (GCS) >13 within 10 minutes after the head trauma, 4) having continued symptoms and signs of concussion one month after the head trauma at the time of neurological examination (e.g. blurred/double vision, vertigo, headache, imbalance, mood/cognitive/sleep abnormalities, convergence insufficiency, eye misalignment, cerebellar/vestibular abnormality, cognitive abnormality as examined by a neurologist/neuro-ophthalmologist (4^th^ author), and 5) having normal hearing. The healthy control group’s inclusion criteria were: 1) being over 15 years of age, 2) have no history of head trauma, ear infection/injury, any psychiatric and/or neurological disorder, and 3) having normal hearing.

Out of 45 referred patients, tested were 38 individuals (19 males, 43 ± 13.5 years) with PCS, out of which 8 were short-term PCS (SPCS- concussion <3 months prior to testing) and 30 patients were long-term PCS (LPCS- concussion >3 months prior to testing). As per International Classification of Headache Disorders-3, post-traumatic headache is called “persistent” if it lasts more than 3 months^44^. Therefore, 3 months is the best reasonable criterion to identify short-term from long-term post-concussion syndrome. That criterion has been extensively used in concussion literature as well.

We also recruited 33 healthy age-and-gender-matched individuals (13 males, 42.5 ± 16.2 years) with no history of concussion as the control group of the study.

The duration between the majority of LPCS patient’s mTBI’s and the recording date was between 5 months to 5 years with the exception for two patients remaining symptomatic for more than 10 years prior to the EVestG recording. The aim was to test PCS patients with ongoing symptomology during (1 week to 3 months) and post (>3 months) normal recovery periods to investigate whether we could identify any malingering or psychogenic symptomology with EVestG assessment. Data were recorded at the Neural Diagnostic Laboratory, Riverview Health Center, Winnipeg, Manitoba. Demographic details of the PCS participants can be found in the supplementary Table S1.

### Signal Analysis

**Figure 1:**
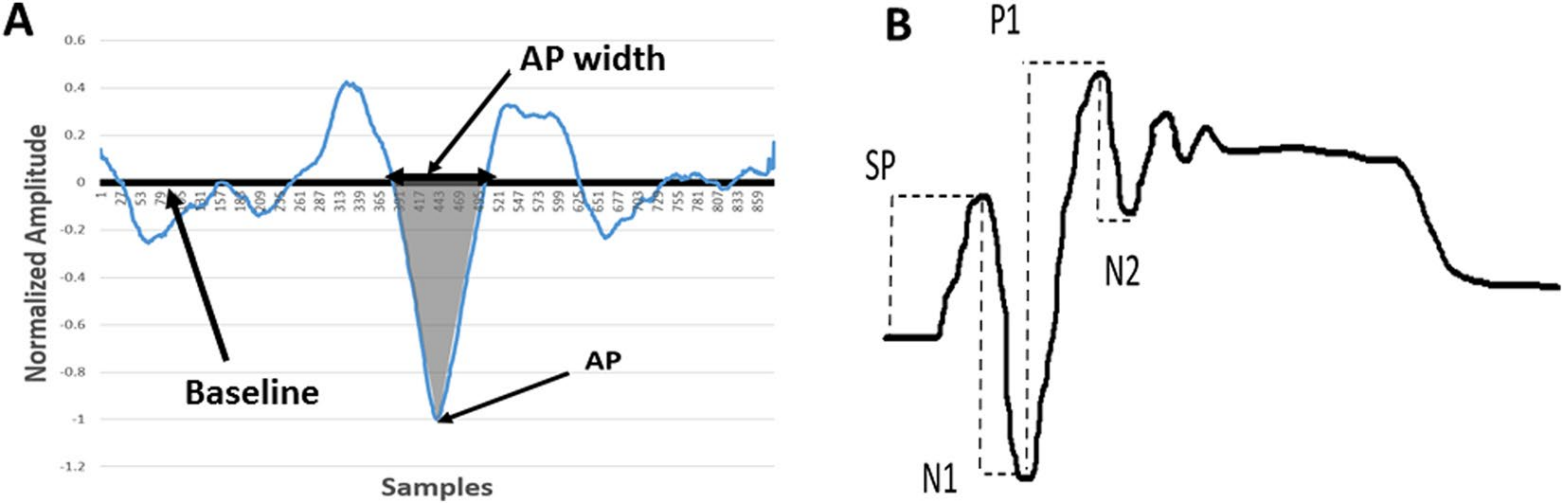
(**A**) A typical normalized field potential (FP). The bounded area between the baseline and the AP (marked area) was used as a characteristic feature. (Horizontal scale 41.6 samples = 1 ms). (**B**) Acoustic compound action potential waveform.

The EVestG-evoked response FPs and their firing pattern from stationary (BGi) segments of the signals were extracted using a wavelet-based signal processing technique called the Neural Event Extraction Routine (NEER)^28^. The muscle artifacts were removed from the recorded signals by a high pass filter set to 300 Hz. Nevertheless, if a signal was corrupted by muscle artifacts, poor electrode placement, movement or contact of the electrodes, that signal was removed from the analysis. Approximately 7% of signals were excluded. The NEER algorithm detects a series of FPs to produce an average FP plot like the one shown in Fig. 1A. The extracted FPs have the same fundamental shape as the vestibular and acoustic compound action potentials^45,46^ (Fig. 1B). However, the cochlear and vestibular periphery have major differences: vestibular axons have a broad spread of thickness and are on average thicker; cochlear axons have a narrow spread and are thinner; this means the averaged vestibular FPs are likely wider than acoustically evoked compound action potentials^47,48^.

Characteristic features of the signals representing the vestibular system and PCS were extracted with the following procedure. Using the FP curve as well as the FP firing pattern, we extracted two types of features as elaborated below.

#### Feature Type 1

After normalizing the FPs with the absolute value of the action potential (AP) point (Fig. 1A), we calculated the area between the baseline and the AP point, which was basically the area of the AP curve below the baseline. This feature type was found significantly different among controls and PCS subjects in a previous study that used a subset of data of this study^42^. However, when we looked at each subject’s AP area individually, we found in some cases there were differences in the descending part of the AP, the ascending part of AP, or on both sides of the AP. The choice of the AP area as our feature can take into account all three differences. Further investigations are needed to see why some have difference in the ascending part while some others in the descending part and some in both parts of the AP.

#### Feature Type 2

**Figure 2:**
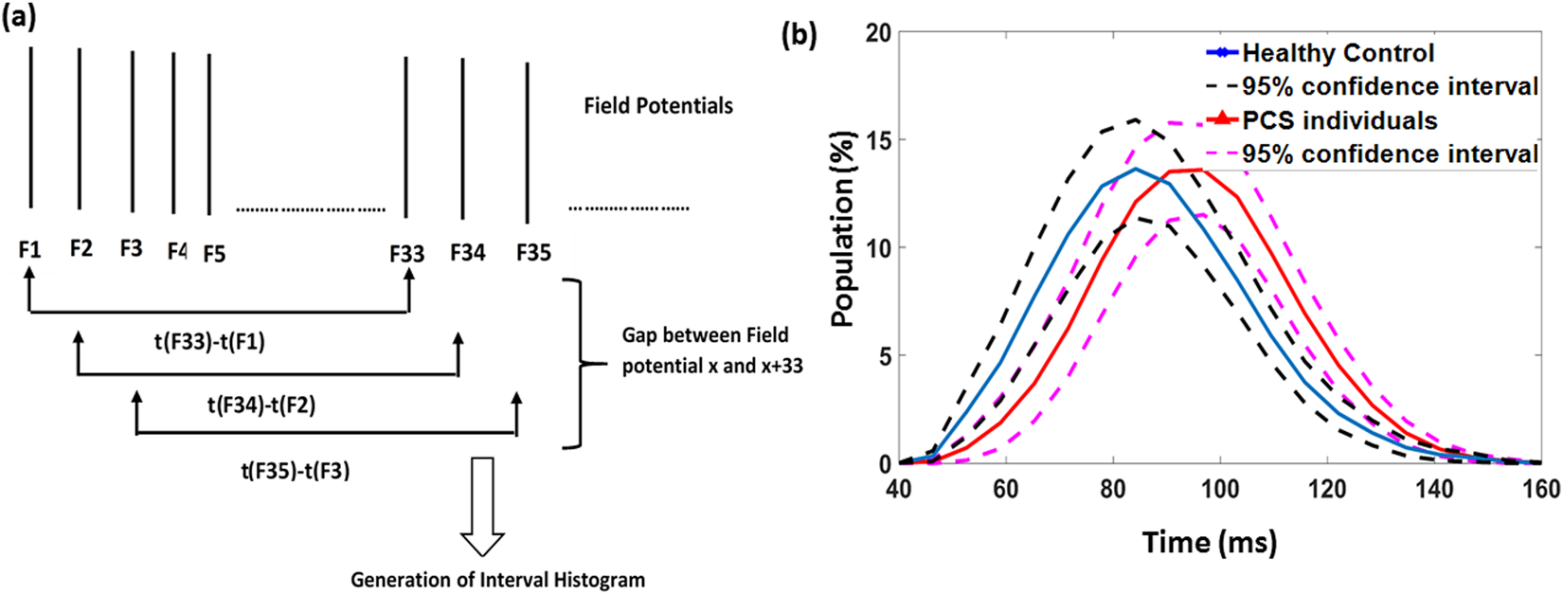
(**a**) The generation process of finding the gap between 33 FPs and generating the interval histogram. (**b**) Interval Histogram for an FP gap equal to 33 FPs during static (no motion) phase (BGi). The blue and red solid lines represent the healthy controls (n = 32) and PCS (long and short-term PCS, n = 38) encased by dashed 95% confidence interval lines respectively.

Beside the FP, the NEER algorithm also provides the time of occurrence of each detected FP. It was shown in^49^ that vestibular efferent spontaneous activity is usually seen in the range 10–50 spikes/s. Thus, we also looked for the low frequency (modulated) spontaneous FP interval activity (~ 10 Hz). Since the average measured time gap that NEER algorithm detects between two FPs is ~ 3.3 ms, a 33 FP gap corresponding to about ~100 ms (10 Hz)^30^ was used (Fig. 2a). Therefore, the average interval histograms based on 33^rd^ (IH33) FP gap during the no movement (BGi) phases from the signals of study participants were generated. A significant (p < 0.05) difference was found between both groups as the average distribution of PCS group was shifted towards the right (lower frequencies and longer gaps) as shown in Fig. 2b. Feature Type 2 comprised the total percentage of the response intervals with bin value more than 90 ms.

**Figure 3:**
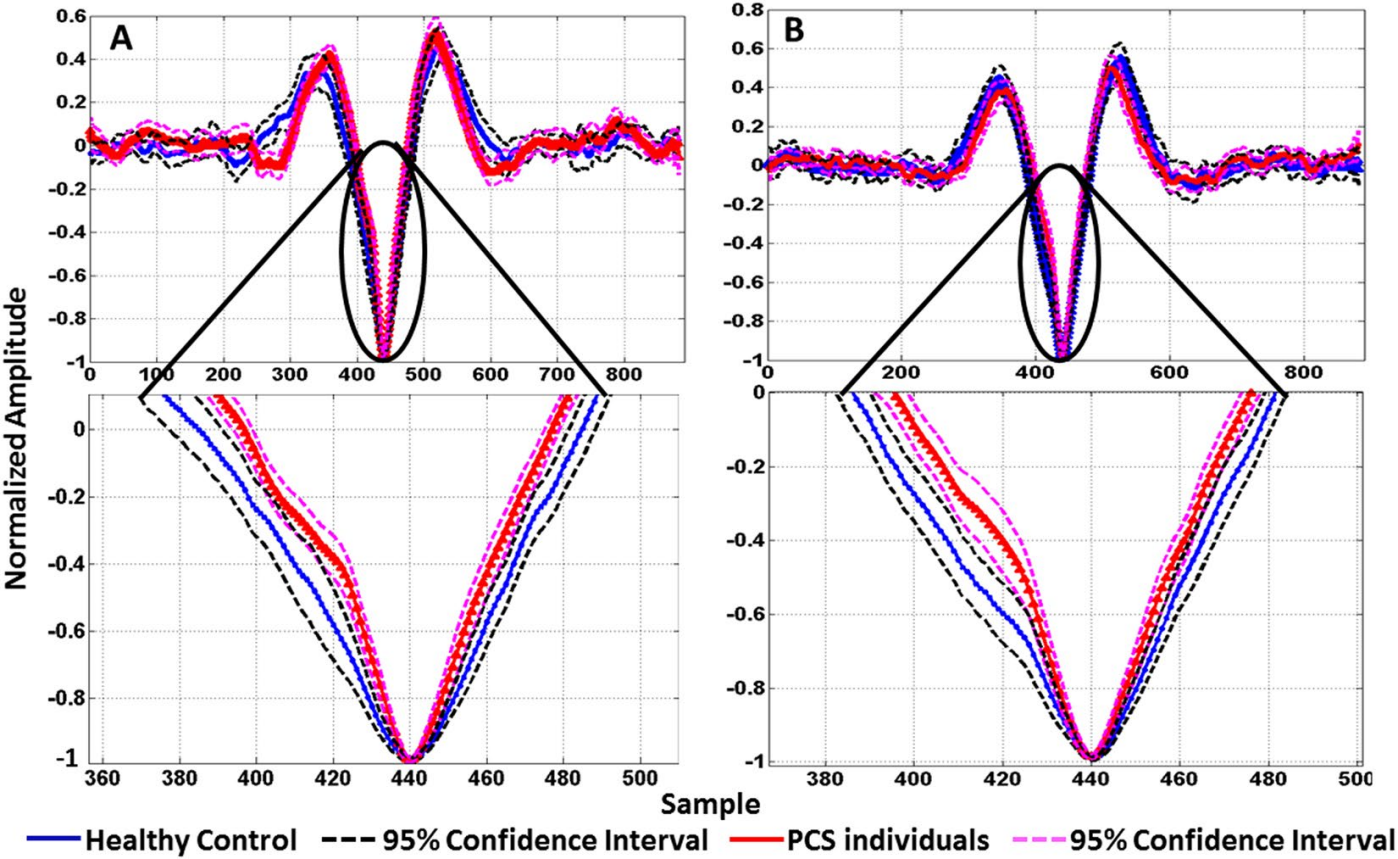
Average response for control (n = 32) and PCS (long and short-term PCS, n = 38) groups. The marked circles/arrows show significant (P < 0.05) difference in the AP area between control and concussed during static segment (BGi) extracted from (**A**) right ear and (**B**) left ear.

Figure 3 shows the mean ± 95% confidence interval of the AP area (Feature Type 1) of the two groups of concussed patients and healthy controls extracted from right (Fig. 3A) and left (Fig. 3B) ears. As can be seen, the averaged AP area of patients with PCS was found smaller than that of the healthy controls. In addition, among all the PCS patients, the ones with SPCS- concussion the AP area was the smallest.

Thirteen out of the 38 PCS participants had a lateral head impact either from left or right. In our previous pilot study^42^, we observed an asymmetry between left and right ear in lateral-impact PCS participants. Indeed, the AP area was always narrower on the coup side, while it was either wider or similar to healthy control response in the contra-coup site. Therefore, we calculated the minimum AP area for left and right ear signals and used this smaller value as a characteristic feature.

Figure 2b shows the average interval histograms of the time between detected 33 FPs for both groups during static phase (BGi). We can see that the concussed histogram is shifted to the right of curve of the healthy controls; this is indicative of an increase in time between IH33 intervals and hypothetically may be related to a reduction or slowing of efferent input. For the same reasons mentioned above on the asymmetry between left and right ear signals for laterally impacted PCS participants, we calculated the maximum IH33 interval difference for left and right ears and used the maximum as our second characteristic feature.

### Classification

We applied linear discriminant analysis (LDA)^50^ as the classification routine for separating healthy controls from concussed patients. LDA is a standard approach for supervised classification; it estimates the membership probability of each class as a Gaussian distribution assuming identical covariance matrices for all classes. Due to small size of our dataset and yet keeping the training and testing separate for an unbiased classification and also to avoid the over-fitting problem, a leave-one-out routine was applied for classification, in which one subject’s data was left out for testing and the rest used as training; this routine was repeated until all subjects were used as test once. In each fold, the two features were tested individually using LDA for classifying the two groups and the resultant accuracy was calculated. The same routine was repeated for the combination of the two features and the resultant accuracy calculated.

Tables 1–4 summarize the LDA classification results with a leave-one-out routine of each of the two features as well as their combination. Feature 1 (the AP area) showed a leave one out test accuracy of 81%, while feature 2 (the IH33) showed a 73% testing leave one out routine accuracy, when each is considered for classifying the groups separately. The combination of the two features increased the leave one out accuracy to 84% with an 81.6% sensitivity and 87.5% specificity (Table 1).

**Table 1:** Testing accuracy, sensitivity and specificity of classification between PCS (n = 38) and Healthy control (n = 32).

	Accuracy (%)	Sensitivity (%)	Specificity (%)	Auc (%)
FEATURE 1	81.42	84.21	78.12	—
FEATURE 2	73.23	73.68	72.72	—
FEATURE 1&2	84.30	81.60	87.50	84.50
FEATURE 1&2 (SVM)*	84.30	88.60	80.00	84.50

LDA classification accuracies using a leave-one-out routine for features 1 and 2 and their combination. Accuracy, Sensitivity and Specificity were calculated. For comparison, SVM classification accuracy for combined features are also presented. AUC represents Area under the ROC curve. *Comparison LDA with SVM.

**Table 2:** Testing accuracy, sensitivity and specificity of classification between LPCS (n = 30) and Healthy control (n = 32).

	Accuracy (%)	Sensitivity (%)	Specificity (%)	Auc (%)
FEATURE 1	77.41	80.00	75.00	—
FEATURE 2	73.01	73.34	72.72	—
FEATURE 1&2	77.41	76.67	78.12	77.40
FEATURE 1&2 (SVM)*	82.30	85.00	80.00	83.7

LDA classification accuracies using a leave-one-out routine for features 1 and 2 and their combination. Accuracy, Sensitivity and Specificity were calculated. For comparison, SVM classification accuracy for combined features are also presented. AUC represents Area under the ROC curve. *Comparison LDA with SVM.

**Table 3:** Testing accuracy, sensitivity and specificity of classification between SPCS (n = 8) and Healthy control (n = 32).

	Accuracy (%)	Sensitivity (%)	Specificity (%)	Auc (%)
FEATURE 1	95.00	87.50	96.87	—
FEATURE 2	87.80	100.00	84.84	—
FEATURE 1&2	95.00	87.50	96.87	92.00
FEATURE 1&2 (SVM)*	97.50	100.00	96.97	93.75

LDA classification accuracies using a leave-one-out routine for features 1 and 2 and their combination. Accuracy, Sensitivity and Specificity were calculated. For comparison, SVM classification accuracy for combined features are also presented. AUC represents Area under the ROC curve.*Comparison LDA with SVM.

**Table 4:** Testing accuracy, sensitivity and specificity of classification between LPCS (n = 30) and SPCS (n = 8).

	Accuracy (%)	Sensitivity (%)	Specificity (%)	Auc (%)
FEATURE 1	76.31	76.67	75.00	—
FEATURE 2	55.26	50.00	75.00	—
FEATURE 1&2	78.95	80.00	75.00	77.50
FEATURE 1&2 (SVM)*	92.42	85.71	93.55	85.83

LDA classification accuracies using a leave-one-out routine for features 1 and 2 and their combination. Accuracy, Sensitivity and Specificity were calculated. For comparison, SVM classification accuracy for combined features are also presented. AUC represents Area under the ROC curve. *Comparison LDA with SVM.

**Figure 4:**
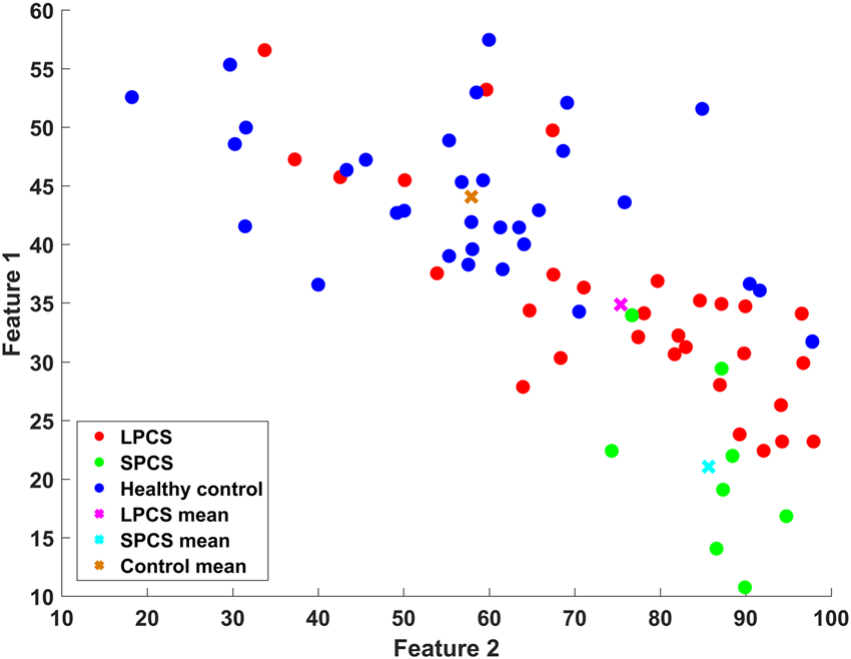
Combination of features 1 and 2 for separating the groups of Control (n = 32) vs. long-term PCS (LPCS- concussion >3 months prior to testing, n = 30) plus short term PCS (SPCS- concussion <3 months prior to testing, n = 8). Feature 1 is the calculated AP area during static phase (BGi). Feature 2 is derived from the IH histogram using a gap equal to 33 FPs.

The two features used are highly correlated (R = −0.7), however we  used the IH33 feature as well as the AP area feature as both are potentially physiologically meaningful features (see discussion). To give a visual representation of the selected features among the two groups of controls and concussed (SPCS and LPCS), the scatter plot of the features for the two groups of concussed and controls are shown in Fig. 4. Additionally, this two-feature combination was also able to separate the SPCS and LPCS subgroups of concussed individuals with a 79% accuracy using a leave one out routine. In addition, the LDA classification accuracy of separating LPCS from healthy control was 77% (Table 2), while its accuracy for separating SPCS from healthy control was 95% (Table 3).

The classification accuracy can be improved using other nonlinear classification techniques or a support vector machine (SVM). However, in small sample size studies, LDA is more reliable than other non-linear classification methods because LDA is more robust to the variance. In other words, if a technique shows a reasonable accuracy using the LDA, then we can be confident that the method will perform better using nonlinear classification methods. For comparison, we did run the classification using SVM. The classification accuracy using SVM for separating LPCS and SPCS groups improved to 92% (Table 4) (c.f. 79% for LDA). Also, SVM classification for separating LPCS from controls improved to 82% (Table 2) (c.f. 77% for LDA), and 97.5% for separating SPCS from controls (Table 3) (c.f. 95% for LDA).

The area under the receiver operating characteristic curve (AUC) was calculated (Tables 1–4) as an additional indicator of the diagnostic ability of the features used in binary classification. The SVM showed a better performance than LDA, especially in for the classification of LPCS and SPCS.

## Discussion

Following an mTBI, the most common symptom after headache is dizziness^51,52^ that can cause balance problems. Poor balance and postural instability have been reported in many studies after mTBI^53–55^ and have been correlated with dysfunction in sensory integration^56,57^. Some symptoms including vertigo and dizziness can be due to neurovascular compression of the 8^th^ cranial nerve^58^. Considering this fact and the link between dizziness and the abnormal function of the vestibular apparatus, the representative results of this study support the hypothesis that vestibular activity is perturbed following mTBI. This study has shown that the EVestG evoked averaged FP responses of the two groups of PCS individuals and healthy controls has classification power to separate PCS individuals from controls.

Vestibular^45^ and acoustic compound action potentials^46,59^ have similar characteristic shape and both are comparable with the extracted vestibular FP of the recorded signals. Many studies discuss the generation of the acoustic compound action potentials (Fig. 1B) and the generation of its different components^46,59–61^, which may help explain the differences seen in the extracted FP between healthy controls and PCS patients in our study. In our results, the significant differences were in the AP part of the FP. According to studies^46,59^, the AP region of the signal corresponds predominantly with the N1 and P1 component (Fig. 1B) of the acoustic compound action potentials. The N1 negative peak is generated by the flow of Na^+^ current through the voltage-gated Na^+^ channels into the primary afferent neuron^46,60^. The P1 component has been shown to be generated by the K^+^ efflux from primary afferent nerve through voltage-gated K^+^ channels^61^. The generation of the P1 peak is still a controversial topic, and it is not clear where it is generated. Some studies have shown that after removing the cochlear nucleus (CN) the P1 peak is reduced^59^. Another study^46^ showed the P1 peak was recovered one hour after removing the CN; this indicates the P1 peak might be produced (at least partially) in the 8^th^ nerve, and may not be entirely of CN origin. Brown *et al*.^46^ showed that applying pressure to the 8^th^ cranial nerve lead to a decrease in the P1 amplitude; that implies any physical change to the 8^th^ cranial nerve results in an AP shape change.

The changes in the AP area observed in the vestibular responses following mTBI in this study can also be partly explained by the fact that after a head trauma, a cascade of neurochemical and neurometabolic events occur^3^. A physical change of the neuronal cell membrane or the axons leads to indiscriminate flux of ions through ion gates. This abnormal process increases the release of the excitatory neurotransmitters like glutamate, resulting in further ionic flux. Then, in order to maintain an ionic balance, the Na/K ATP-dependent channels become activated, and that increases the glucose metabolism. This mismatch of energy supplies and demand leads to further cell injury and dysfunction^3^. Linking the process of the development of the AP after stimulation with the generation of N1 and P1 peaks of the acoustic compound action potentials (Fig. 1B), the abnormal movement of the ions in and out the cell membrane would produce a change the AP shape. However, this change is likely to be short-term, lasting less than 3 months.

To explain our results supporting of the long-term symptomatology of our PCS participants, we need to consider the possibility of a more permanent damage that might have occurred in the 8^th^ nerve region. In studies^62,63^ it was shown that when axons were loaded *in vitro*, the sodium gates became perturbed resulting in the sodium influx and subsequent depolarization with calcium influx through voltage-sensitive calcium channels and the reversal of the sodium-calcium exchangers. They also showed that post mechanical trauma and deformation of the axons triggered Na^+^ influx through sensitive voltage-gated sodium channels (NaChs); that would result in an increase in Ca^+2^ influx and subsequent proteolysis of the NaCh α-subunit. As a result of the α-subunit degradation, the α-subunit promotes persistent elevation in Ca^+2^, which helps explain the narrower AP area in long-term PCS. In another study^64^ plaques composed of amyloid β (Aβ) were found in the damaged axons following a brain trauma in humans. In the same study, the authors described the accumulation of Aβ in the damaged axons as well as in a limited number of neurons of the cortex, hippocampus and cerebellum 3 and 7 days and 6 months after trauma in the TBI mice. As mentioned before, the extracted FP curve was always narrower for PCS individuals compared to healthy controls. We speculate that this change is due to an increase efflux and influx of sodium-potassium ions that might be explained by the accumulation of intra-axonal Ca^+2^ ^62–65^. The ability of the Aβ to form neurofibrillary tangles could be the consequence of its ability to increase the Ca^+2^ influx into neurons^65^.

Following Brown’s^46^ observation that the generation of N1 and P1 peaks of the acoustic compound action potentials were entirely by the 8^th^ nerve, we believe the changes we observe in the AP area (Fig. 3A,B) of the PCS individuals arise from the 8^th^ cranial nerve changes. The difference in the AP area during a static phase was observed to be significantly different between both groups, i.e. narrower. This indicates that the PCS individuals’ vestibular system responded differently from that of the healthy controls. The difference in the AP area among the two groups was observed in both peaks (N1 and P1); this implies more influx of the Na^+^ and more K^+^ efflux in the PCS group compared to those of the healthy controls, which in turn implies a difference in the depolarization and repolarization mechanism of both groups.

Using the two-feature combination, there were seven misclassified PCS patients. It is of interest to note that most (5 out of 7) of the misclassified patients had the head trauma more than 1.5 years prior to our recording. Thus, considering the plasticity factor of the nerves, one may speculate that those patients had more time to recover. However, one of the two patients who had the impact more than 10 years prior to recording, was classified as healthy, while the other patient was classified as PCS. This suggests PCS individuals’ symptoms may persist for a long time and recovery depends on their brain’s plasticity or other confounding influences like anxiety.

Accurate diagnosis and prognosis of the TBI consequence are essential for patient care and long-term rehabilitation. In a recent study^24^, using a robotic-assisted assessment of neurological function, they investigated if PCS following mTBI can be predicted during the initial presentation to an emergency department. However, they only validated their prediction accuracy over a short (3 weeks post-injury) duration.

In another study, using qEEG, it was claimed that qEEG analysis, independent of other assessments, could predict the severity of the injury with high accuracy in a post-trauma period ranging from months to 8 years^22,23^. However, qEEG has not shown a clear ability to differentiate between SPCS and LPCS^22,23^ as both SPCS and LPCS show increased delta and reduced alpha band power^27^, while PCS within the recovery time and recent to the injury is expected to be different than PCS beyond the recovery time. The authors in^27^ continue, “*EEG/qEEG findings in mTBI have been hypothesized to be related to the known pathophysiology of mTBI, and in some cases have also been corroborated with other investigations such as neuroimaging or histopathology*” but also state, “*Although the literature indicates the promise of qEEG in making a diagnosis and indicating prognosis of mTBI, further study is needed to corroborate and refine these methods*”^27^. In other study^25^ it was indicated that qEEG provides, at best, an imperfect assessment of mTBI and reports the high specificity of qEEG evaluations of TBI must be interpreted with care questioning qEEG’s disease specificity. However, these authors also state “*The published literature does indicate, however, that it (qEEG) can be an important complement to other assessment procedures*”^25^.

In this current study, we showed the use of a quantitative physiological measure of the vestibulo-acoustic response (EVestG signals) has promising potential to identify SPCS and LPCS both from each other and from healthy control populations. EVestG is shown to have the potential to monitor the PCS within the non-persisting symptom recovery time and also differentiate it from persistent PCS beyond the normal recovery time. However, according to the literature, the recovery time might be within the first days, weeks, up to 3 months after the injury. Thus, it is possible that some of our SPCS participants were beyond the non-persisting symptom recovery time, and appeared only with persistent symptoms (which may be a reduced form of those in LPCS). That may explain why two out of eight SPCS were classified in the LPCS group (Fig. 4). This is considered a study limitation. The main limitations of this study are: 1) The overall sample size, particularly the SPCS sample size, was small; 2) There were 3 times as many LPCS as SPCS; and 3) Detailed symptomatology was not recorded and graded at regular time intervals. Future studies should address these issues. Overall, the results of this study suggest the AP area of the responses during static phase is a promising feature with a sensitivity to post-concussion symptoms. The results of this study are encouraging the use of EVestG analysis for screening and monitoring PCS patients (SPCS and LPCS) and the recovery from LPCS-concussion.

### Methodology

Data used in this study was collected through two separate but related projects. One was to investigate the feasibility of the EVestG technology for diagnosis of concussion, and the second was a clinical trial (ClinicalTrials.gov Identifier: NCT02426749) for treatment and recovery monitoring (using EVestG) of post TBI Syndrome. Both studies were approved by the University of Manitoba Biomedical Research Ethics Board, and all the participants signed an informed consent prior to the experiment. All experimental procedures were performed in accordance with the protocol approved by the Biomedical Research Ethics Board and its regulations.

### EVestG Recording Procedure

1. Placing the electrodes: the ear canal wick electrode was placed in each ear canal close to the ear drum (TM-EcochGtrode, Bio-logic, France (Fig. 5.A)). Identical reference electrodes were placed on each ipsilateral ear lobe close to the ear canal (Fig. 5B). One common ground (Biopac EL258S) electrode was placed on the forehead (Fig. 5C).

**Figure 5:**
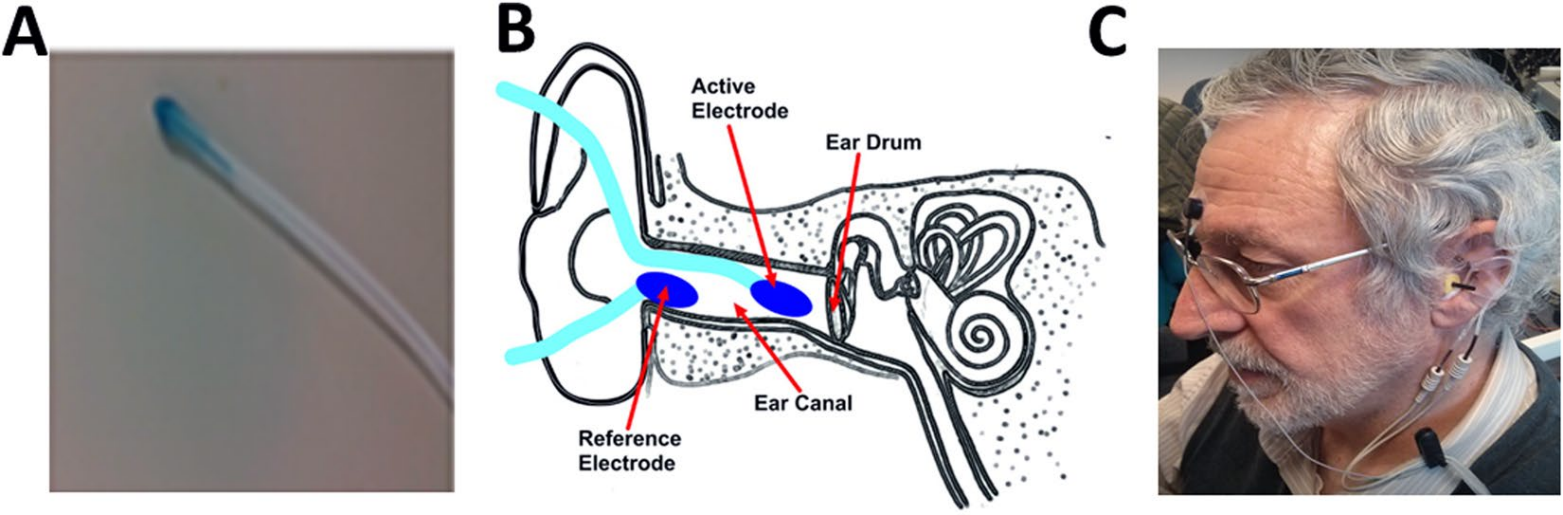
(**A**) Ear electrode; (**B**) electrodes placement; (**C**) participant connection.
2. After placing the electrodes, the participant was positioned in an acoustically attenuated (>30 dB) and electromagnetically shielded chamber, and seated in a stationary hydraulic chair, with their head supported by a headrest (Fig. 5C). Participants were instructed to close their eyes closed during the recordings.
3. The signals of both ears were recorded using Spike2^TM^ with a sampling rate of 41,666 Hz for compatibility with previous studies.

The vestibulo-acoustic system is highly spontaneously active^32–34^. In this study, in order to minimize any artifacts caused by body movement which may corrupt the recorded signal and to, at least initially, consider the ability of features based only on the spontaneous activity of the vestibule-acoustic system to discern PCS, the analyzed EVestG recorded signals are only from the stationary phase (BGi) recordings^28^. The analyzed segment was the average of three BGi recordings.

### Data Availability

The data that support the findings of this study are available from Neural Diagnostics Pty. Ltd. but restrictions apply to the availability of these data, which were used under license for the current study, and so are not publicly available. Data are however available from the authors upon reasonable request and with permission of Neural Diagnostics Pty. Ltd.

## Electronic supplementary material

Supplementary Information
