# Author Correction: Quantitative measurement of post-concussion syndrome Using Electrovestibulography

**DOI:** 10.1038/s41598-020-59901-8

**Published:** 2020-02-14

**Authors:** Abdelbaset Suleiman, Brian Lithgow, Zeinab Dastgheib, Behzad Mansouri, Zahra Moussavi

**Affiliations:** 1 Biomedical Engineering Program, University of Manitoba, Winnipeg, MB Canada; 2 Department of Internal Medicine (Neurology), University of Manitoba, Winnipeg, MB Canada; 3 Monash Alfred Psychiatry Research Center, Monash University, Melbourne, Australia

Correction to: *Scientific Reports* 10.1038/s41598-017-15487-2, published online 27 November 2017

The original version of this Article contained a typographical error in the spelling of the author Zeinab Dastgheib, which was incorrectly given as Zeinab Dastghieb. This has now been corrected in the PDF and HTML versions of the Article.

