# Supplementary material for: Quantitative measurement of post-concussion syndrome Using Electrovestibulography
Source: Sci Rep. 2017 Nov 27;7:16371. doi: 10.1038/s41598-017-15487-2 (PMC5703984; doi:10.1038/s41598-017-15487-2)
Supplement: Supplementary file 1 — Supplementary Information [file 41598_2017_15487_MOESM1_ESM.docx]

Supplementary Table S1: A list of Post-concussion syndrome participants' demographics

| Patient ID | Patient  Code | Age | Gender | Hand | Time between impact and EVestG recording | GCS | Impact site | MADRS | Syndromes |
| --- | --- | --- | --- | --- | --- | --- | --- | --- | --- |
| 1 | Anpl | 49 | Female | R | 9 m | 15 | Lateral- head | 4/60 Normal | 1. Headache 2. Dizziness |
| 2 | ArKr | 23 | Male | R | 1yr,5m | 15 | Forehead | 4/60 Normal | 1. Dizziness 2. Memory loss 3. focusing |
| 3 | BeKo | 21 | Male | L | 7m | 14 | Lateral- head | 5/60 Normal | 1. headache 2. memory loss 3. dizziness 4. sleep disturbance 5. sensitivity to light |
| 4 | DoBu | 56 | Female | L | 3yr,10m | 13 | Lateral- head | 25/60 Moderate Depression | 1. headaches 2. dizziness 3. blurry vision |
| 5 | GaMa | 51 | Male | R | 1yr | 15 | Top- head | 10/60 Mild Depression | 1. headaches 2. dizziness 3. blurry vision |
| 6 | GeGa | 61 | Female | R | 1yr,10m | 14 | Back-head | 27/60 Moderate Depression | 1. headaches 2. dizziness |
| 7 | IvBh | 46 | Female | R | 3m | 15 | Back- head | 4/60 Normal | 1. headaches 2. dizziness 3. blurry vision 4. sensitivity to noise |
| 8 | KaBu | 43 | Female | R | 1yr,11m | 15 | N/A* | 5/60 Normal | 1. headaches 2. dizziness 3. blurry vision 4. memory loss |
| 9 | LaJo | 51 | Male | R | 19yrs | 15 | Back-head | 26/60 Moderate Depression | 1. headaches 2. dizziness 3. blurry vision 4. memory loss |
| 10 | LuSm | 22 | Female | R | 3yrs | 15 | Lateral- head | 24/60 Moderate Depression | 1. headaches 2. dizziness 3. blurry vision 4. memory loss 5. sleep disturbance |
| 11 | MeKa | 34 | Male | R | 9m | 15 | Forehead | 1/60 Normal | 1. headaches 2. dizziness 3. blurry vision |
| 12 | MiJo | 52 | Female | R | 8m | 15 | Top-head | 18/60 Mild Depression | 1. headaches 2. dizziness 3. concentration |
| 13 | RoAl | 36 | Male | R | 8m | 15 | Lateral- head | 26/60 Moderate Depression | 1. dizziness |
| 14 | RoFr | 39 | Female | R | 2yrs,1m | 15 | Lateral-head | 6/60 Normal | 1. headaches 2. dizziness |
| 15 | ShSh | 34 | Male | R | 2m | 13 | N/A* | 17/60 Mild Depression | 1. headaches 2. dizziness 3. blurry vision 4. sensitivity to light/noise |
| 16 | SiGi | 51 | Male | L | 2yrs,6m | 15 | Lateral-head | 30/60 Moderate Depression | 1. headaches 2. dizziness 3. blurry vision 4. longer to think |
| 17 | StAr | 21 | Male | R | 2yrs | 15 | Forehead | 8/60 Mild Depression | 1. headaches 2. dizziness 3. blurry vision |
| 18 | StTi | 50 | Female | L | 1yr,6m | 15 | Top/back-head | 26/60 Moderate Depression | 1. headaches 2. dizziness 3. sensitivity to noise |
| 19 | SuSi | 40 | Female | R | 7m | 15 | Lateral-head | 4/60 Normal | 1. headaches 2. dizziness |
| 20 | TaDr | 46 | Female | R | 5yrs | 15 | N/A* | 8/60 Mild Depression | 1. headaches 2. dizziness 3. memory loss 4. concentration |
| 21 | MaSt | 47 | Female | L | 8m | 15 | Forehead, Back-head and Lateral-head** | 5/60 Normal | 1. headaches 2. dizziness 3. blurry vision 4. nausea |
| 22 | AnBa | 50 | Female | R | 5m | 15 | Back-head | 21/60 Moderate Depression | 1. headaches 2. dizziness 3. sensitivity to light 4. sleep disturbance 5. memory loss 6. blurry vision |
| 23 | JaSo | 30 | Male | R | 4yrs | 14 | N/A* | 15/60 Mild Depression | 1. headaches 2. Sensitivity to light |
| 24 | switbi001 | 36 | Male | R | 2w | 15 | Lateral-head | 5/60 Normal | 1. headaches 2. dizziness 3. blurry vision 4. nausea |
| 25 | switbi002 | 50 | Male | R | 2w | 15 | Lateral-head | 3/60 Normal | 1. headaches 2. dizziness 3. memory loss |
| 26 | PhSt | 60 | Male | L | 2w | 15 | Lateral-head | 1/60 Normal | 1. headaches 2. dizziness 3. sensitivity to light |
| 27 | FrDu | 67 | Female | R | 14yrs,8m | 14 | N/A* | 6/60 Normal | 1. Memory loss 2. Concentration 3. Blurry vision |
| 28 | KrPe | 40 | Male | R | 1w | 15 | Forehead | 6/60 Normal | 1. Headaches 2. Nausea |
| 29 | KyPa | 28 | Female | R | 1yr,9m | 15 | Back-head | 6/60 Normal | 1. Headaches 2. Balance |
| 30 | GiEs | 43 | Male | R | 7m | 15 | Forehead | 28/60 Moderate Depression | 1. Headaches 2. Balance problems 3. Sleep disturbance 4. Blurry vision |
| 31 | ChSt | 46 | Female | R | 6m | 13 | Top-head | 23/60 Moderate Depression | 1. Headaches 2. Balance problems 3. Blurry vision |
| 32 | MaPa | 26 | Female | R | 1yr,9m | 15 | Top-head | 10/60 Mild Depression | 1. Headaches 2. Balance problems 3. Blurry vision |
| 33 | NiLe | 19 | Male | R | 1m | 15 | N/A* | 5/60 Normal | 1. Headaches 2. Concentration |
| 34 | RaFo | 71 | Male | R | 3yrs | 15 | Back-head | 8/60 Mild Depression | 1. Headaches 2. Fatigue 3. Blurry vision |
| 35 | ShMo | 64 | Female | R | 1yrs | 14 | Lateral-head | 10/60 Mild Depression | 1. Headaches 2. Concentration 3. Blurry vision 4. Balance problem |
| 36 | ScCa | 25 | Male | R | 1m | 15 | Forehead | 6/60 Normal | 1. Headaches 2. Dizziness 3. concentration |
| 37 | BeGi | 63 | Male | R | 3m | 15 | Lateral-headpp | 4/60 Normal | 1. Headaches 2. Noise sensitivity 3. Light sensitivity 4. dizziness |
| 38 | PaHi | 78 | Female | R | 3m | 14 | Back-head | 5/60 Normal | 1. Dizziness 2. Fatigue 3. Blurry vision 4. Double vision |

*site of the impact is unknown.

** Participant experienced three Impacts.
